# Unintended Prolonged Opioid Use: Protocol for a Case-Controlled Trial

**DOI:** 10.2196/72032

**Published:** 2025-03-24

**Authors:** W Michael Hooten, Darin J Erickson, Marek Chawarski, Natalie A Scholz, Jennifer F Waljee, Chad M Brummett, Molly M Jeffery

**Affiliations:** 1 Division of Pain Medicine Department of Anesthesiology and Perioperative Medicine Mayo Clinic Rochester, MN United States; 2 Division of Epidemiology and Community Healty University of Minnesota Minneapolis, MN United States; 3 Department of Emergency Medicine Department of Psychiatry Yale University New Haven United States; 4 Department of Surgery Indiana University Bloomington United States; 5 Department of Anesthesiology University of Michigan–Ann Arbor Ann Arbor United States; 6 Division of Health Care Delivery Research Department of Emergency Medicine Mayo Clinic Rochester, MN United States

**Keywords:** opioid use, case-control, unintended opioid use, prolonged opioid use, prospective

## Abstract

**Background:**

Misuse of prescription opioids remains a public health problem. Appropriate short-term use of these medications in opioid-naive patients is indicated in selected settings but can result in unintended prolonged opioid use (UPOU), defined as the continuation of opioid therapy beyond the period by which acute pain would have been expected to resolve. Clinical strategies aimed at preventing UPOU are lacking due to the absence of information about how this poorly understood clinical phenomenon actually develops.

**Objective:**

In this research project, 3 Clinical and Translational Science Awards (CTSA) programs (Mayo Clinic, University of Michigan, and Yale University) leveraged the conceptual framework for UPOU to investigate how patient characteristics, practice environment characteristics, and opioid prescriber characteristics facilitate or impede UPOU. All data management and analyses were conducted at a fourth CTSA program (University of Minnesota). This work was accomplished by pursuing 3 specific aims.

**Methods:**

In aim 1, opioid-naive adults receiving an initial opioid prescription were recruited for study participation. Opioid prescriptions were identified longitudinally, and patterns of use were categorized as short-term, episodic, or long-term use using established criteria. Using a prospective case-control design, patients progressing to UPOU were matched 1:1 with patients who did not develop UPOU, and differences in patient characteristics were assessed. In aim 2, clinicians who prescribed opioids to patients in aim 1 were identified and recruited for prospective assessments. Institutional and individual practice environments were assessed using a validated self-report survey. In aim 3, structural equation modeling was used to evaluate data collected in aims 1 and 2, and identified interactions were further evaluated in a large national administrative claims database.

**Results:**

Patient recruitment began on August 1, 2019. However, due to the COVID-19 pandemic, patient recruitment was slowed and intermittently interrupted over the ensuing 3-year period. As a result of regional variations in the impact of the COVID-19 pandemic on research activities, the majority of patient and clinician recruitment occurred at the Mayo Clinic site.

**Conclusions:**

Following complete data analyses, it is anticipated that electronic health record systems will be leveraged to help clinicians identify at risk patients and to develop direct-to-patient educational materials to raise awareness of the risk factors for developing UPOU.

**Trial Registration:**

ClinicalTrials.gov NCT04024397; https://clinicaltrials.gov/study/NCT04024397

**International Registered Report Identifier (IRRID):**

DERR1-10.2196/72032

## Introduction

### Background

Misuse of prescription opioids remains a public health problem [1]. Appropriate short-term use of these medications in opioid-naive patients is indicated in selected settings but can result in a previously underrecognized segue to unintended prolonged opioid use (UPOU), defined as the continuation of opioid therapy beyond the period by which acute pain would have been expected to resolve [2,3]. Clinical strategies aimed at preventing UPOU are lacking due to the absence of information about how this poorly understood clinical phenomenon actually develops.

**Figure 1:**
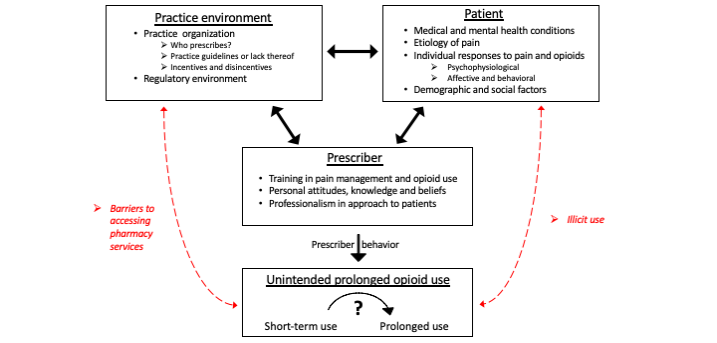
The conceptual framework for unintended prolonged opioid use.

Investigators at Mayo Clinic previously organized a group of thought leaders to develop a conceptual framework for understanding the broad array of factors potentially contributing to UPOU [4]. A conceptual framework is essential both to guide the study of this clinical problem and to identify potential targets for interventions aimed at mitigating the development of UPOU. The framework is comprised of 3 domains, including patient characteristics, practice environment characteristics, and opioid prescriber characteristics that interact to either facilitate or impede UPOU (Figure 1) [4].

Within each domain, potential factors, drawn from the relevant literature, moderate or mediate the influence of each domain. However, the necessary evidence within each of these domains is frequently lacking. This is critically important because 25% of patients in a population who received an initial opioid prescription proceeded to UPOU [3]. This work relied on a medical records linkage system unique to the geographically defined region, limiting the ability to perform larger studies across different patient populations. It also did not perform prospective assessment of several factors important to evaluate the proposed conceptual framework.

### Study Objectives

In this research project, 3 Clinical and Translational Science Awards (CTSA) programs (Mayo Clinic, University of Michigan, and Yale University) leveraged the conceptual framework for UPOU to investigate how the patient characteristics, practice environment characteristics, and opioid prescriber characteristics facilitate or impede UPOU. All data management and analyses were conducted at a fourth CTSA program (University of Minnesota). This work was accomplished by pursuing three specific aims.

#### Specific Aim 1

The first aim was to identify incident cases of UPOU and prospectively assess their characteristics in comparison to new opioid users who did not progress to UPOU. At each site, opioid-naive adults receiving opioid prescriptions were enrolled. Opioid prescriptions and self-reported opioid use were followed, and patients progressing to UPOU were identified in real time. A matched sample of patients who did not develop UPOU were recruited for assessment of framework elements, including biochemical confirmation of opioid use, pain-related measures of physical and emotional functioning, and medical and social histories.

#### Specific Aim 2

The second aim was to assess clinicians prescribing opioids to incident cases of UPOU and new opioid users who did not progress to UPOU. Clinicians treating patients recruited in aim 1 were recruited for prospective assessment of prescriber characteristics including questions about past training in pain management, and attitudes and beliefs about pain and opioid use. The practice environment was assessed including practice organization, practice size, and estimated proportion of patients receiving pain management services.

#### Specific Aim 3

The third aim was to evaluate the conceptual framework. Using the information gathered in aims 1 and 2, structural equation modeling (SEM) was used to evaluate the associations between framework elements in each domain. Identified interactions were further evaluated in a large nationally representative administrative claims database.

## Methods

### Study Settings and Participants

#### Overview

In specific aim 1, opioid-naive adults receiving an opioid prescription (n=780 at each site) were identified at each clinical site (Mayo Clinic, University Michigan, Yale University) and recruited for study participation. A research coordinator assisted in activating a mobile platform on each patient’s personal smartphone for purposes of providing informed consent and completion of study outcome measures. Opioid prescriptions were identified longitudinally by review of each patient’s electronic health record (EHR). Patients progressing to UPOU were identified in real time, and patterns of opioid use were categorized as short-term, episodic, or long-term use using the Consortium to Study Opioid Risks and Trends (CONSORT) criteria [5]. Patients meeting criteria for episodic or long-term use were considered to have progressed to UPOU. Characteristics associated with UPOU were used to assess framework elements, including biochemical confirmation of opioid use; pain-related measures of physical and emotional functioning; and medical, surgical, psychiatric, and social histories.

In specific aim 2, clinicians who prescribed opioids to patients in aim 1 were identified and recruited for prospective assessments. Institutional and individual practice environments were assessed using a self-report survey validated in a national sample of physicians [6].

In specific aim 3, SEM was used to evaluate data collected in aims 1 and 2 to identify the associations between framework elements in each domain. Identified interactions were further evaluated in a large national administrative claims database (OptumLabs Data Warehouse [OLDW]).

#### Study Settings

Each CTSA clinical site leveraged established resources and clinical infrastructure.

#### Mayo Clinic

Olmsted County residents who previously consented to have their medical records used for research purposes were identified using the Rochester Epidemiology Project (REP) medical records linkage system [7,8]. The REP provides access to all medical records for Olmsted County residents. The indications for opioid use in these patients were both surgical and nonsurgical pain. Patients recruited from this site reflected the characteristics of UPOU as it developed in a geographically defined population.

#### University of Michigan

The surgical specialty clinics at the University of Michigan were used to identify opioid-naive patients receiving an initial opioid prescription following common surgical procedures, including knee and hip arthroplasty, inguinal hernia repair, intra-abdominal procedures, and thoracic and breast procedures [9]. The indication for opioid use in these patients was acute surgical pain. Patients recruited from this site reflected the characteristics of UPOU as it developed in a surgical setting.

#### Yale University

Yale New Haven Health is a large health care delivery system that provides services to individuals residing in the greater New Haven, Connecticut area. The EHR was leveraged to identify opioid-naive adults receiving an initial opioid prescription. These individuals were contacted using the patient communication portal of the EHR to assess their interest in participating in the research project. The indication for opioid use among these patients was surgical and nonsurgical pain. Patients recruited from this site reflected the characteristics of UPOU as it developed in a large health care delivery system.

#### Study Participants

A total of 780 patients were approached for recruitment at each site (N=2340), and eligibility criteria for study participation are outlined in Textbox 1. Patients meeting these eligibility criteria were further categorized based on CONSORT criteria [5]. Criteria for the long-term CONSORT category included episodes of opioid prescribing lasting longer than 90 days and including 120 or more total days of supply or 10 or more prescriptions [5]. Criteria for the episodic CONSORT category included episodes lasting 90 days or longer, with total days of supply being fewer than 120 and the total number of prescriptions filled being fewer than 10. Criteria for the short-term CONSORT category included episodes of opioid prescribing lasting 90 days or fewer [5]. Although study participation was limited to adults who own a smartphone, recent data from January 2018 demonstrated 77% of US residents own a smartphone [10].

**Eligibility criteria.**

**Inclusion criteria:**

- Age ≥18 years
- No use of opioids for 6 months before the issuance of the initial opioid prescription as confirmed by review of the electronic health record and patient self-report
- Willingness to participate in all aspects of the study including use of the mobile platform on their personal smartphone

**Exclusion criteria:**

- Cancer-associated pain
- Concurrent treatment for cancer (eg, chemotherapy and radiation therapy)
- Residence in an extended care facility
- Any surgery or hospitalization within the past 6 months
- Mental health disorders that could impede functioning in an ambulatory care setting (eg, schizophrenia and dementia)
- Non–English-speaking individuals
- No smartphone; although study participation was limited to adults who own a smartphone, recent data from January 2018 demonstrated that 77% of US residents own a smartphone [10]

### Specific Aim 1

#### Overview

At each of the 3 sites, a total of 780 opioid-naive adults receiving an initial opioid prescription were approached for enrollment. Subsequently issued opioid prescriptions were monitored by reviewing the EHR, and patients progressing to UPOU were identified in real time. Time-matched samples of patients who did and did not develop UPOU were recruited for assessment of outcome measures.

#### Participant Recruitment

Participant recruitment was site specific. For example, the Mayo Clinic site leveraged the resources of the REP to identify a population-based cohort of potential patients who had previously provided informed consent for the use of their medical records for research purposes. Since 2002, Mayo Clinic and Olmsted Medical Center have used a proprietary software system to document and manage all prescriptions including prescriptions for opioids [11]. These two institutions provide a vast majority of medical care for Olmsted County residents [7,8,12,13]. The electronic prescription system was used to identify previously opioid-naive Olmsted County residents receiving an initial opioid prescription as previously described [3]. These individuals were contacted by telephone and invited to participate in the study. The Michigan site used resources associated with the University of Michigan Analgesic Outcomes Study (AOS) [9]. The AOS is a prospective, observational cohort registry of postsurgical acute and chronic pain outcomes. Previously opioid-naive patients in the AOS registry were invited to participate in study. The Yale University site used the EHR to identify previously opioid-naive patients receiving an initial opioid prescription. More specifically, the EHR was screened by study personnel for the issuance of any opioid prescription, and previously opioid-naive patients were contacted using the EHR patient portal system and invited to participate in the study.

#### Mobile Smartphone Platform

All study participants were assisted by study personnel in downloading the smartphone platform (CareEvolution). The smartphone platform complied with the security and privacy controls defined by the National Institute of Standards and Technology SP 800 53 Rev 5 as designated by the Federal Information Security Modernization Act. The platform was granted authorization to operate by the National Institutes of Health and is Health Insurance Portability and Accountability Act compliant [14]. The smartphone platform was used to obtain informed consent and to collect all outcome measures.

#### Timeline for Establishing Consortium to Study Opioid Risks and Trends Criteria for Opioid Use

**Figure 2:**
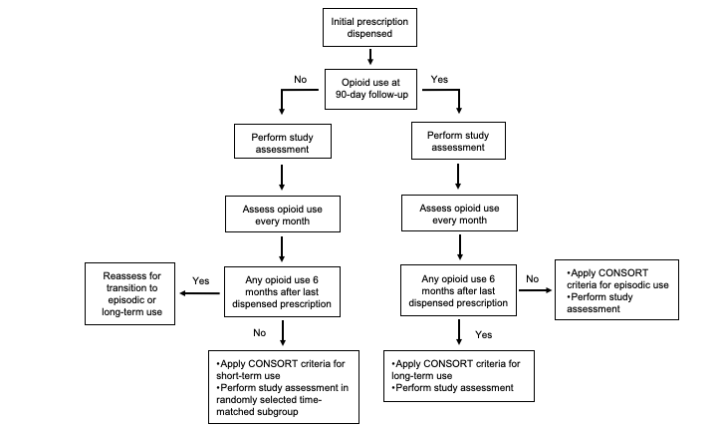
Flow diagram for establishing Consortium to Study Opioid Risks and Trends (CONSORT) criteria for opioid use.

The timeline of the study was governed, in part, by the temporal requirements for applying CONSORT criteria (Figure 2). The beginning date of an episode of opioid use was defined as the date that the initial prescription was issued, with no previous opioid prescriptions issued for the preceding 6-month time period. The end date of an episode was the date that the last dispensed medication supply was exhausted, based on the days of supply as documented in the prescription instructions with no opioid dispensing in the ensuing 6 months. Patients who continued to use opioids on day 90 following issuance of the initial opioid prescription were invited to participate in the study assessment. Although it was not possible to accurately apply CONSORT criteria for episodic or long-term use on day 90, the study assessment was performed at this time point to ensure patients were captured at the earliest stages of episodic or long-term use. The status of opioid use in these patients was monitored every month by reviewing the EHR. This approach to follow-up limits the time interval between establishment of CONSORT criteria for episodic or long-term use and the final study assessment.

A randomly selected group of patients meeting criteria for short-term use at day 90 were invited to participate in the study assessment. This group of patients was selected to be time-matched 1:1 to patients continuing to use opioids at day 90 (initial issue date ±2 weeks) Although it was not possible to accurately apply CONSORT criteria for short-term use until month 6 following issuance of the last prescription, the study assessment was performed at this time point to ensure that patients were captured in the earliest stages of short-term use. Because it was anticipated that the majority of the cohort would meet criteria for short-term use, it was not feasible to invite all patients to participate in the study assessment.

#### Study Assessments

Assessments were performed at 2 time points: 3 months following dispensing of the initial opioid prescription and 9 months following dispensing of the initial opioid prescription.

#### Demographics and Clinical Characteristics

Demographic and clinical characteristics were assessed including age, sex, ethnicity, marital status, education, employment status, BMI, and past medical and surgical histories including chronic pain.

#### Verification of Opioid Status and Dose

Opioid use and dose were verified by review of pharmacy and medical records. Opioid doses were converted to daily morphine equivalents using a conversion calculator as previously reported [15-17]. The opioid status was biochemically verified with a urine toxicology screen.

#### Occurrence and Risk of Opioid Misuse

The occurrence and risk for opioid misuse was assessed using 3 instruments. The Alcohol, Smoking, and Substance Involvement Screening Test (ASSIST) was administered. The ASSIST is validated [18] and used in the National Institute on Drug Abuse Quick Screen [19]. The Prescribed Opioid Difficulties Scale [20] was used to assess for substance use disorders associated with use of prescribed opioids [21]. The Opioid Risk Tool is a brief questionnaire that is validated [22] and was used to assess the risk of opioid misuse.

#### Symptoms of Opioid Withdrawal

Symptoms of opioid withdrawal were assessed using the Clinical Opiate Withdrawal Scale, which is an observer-rated instrument [23,24].

#### Opioid Craving

Opioid craving was assessed using responses (0 to 100 on a visual analog scale) to 3 questions: (1) How much do you crave opioids? (2) How often do you think about the next dose? and (3) How strong is your urge to take more medication than prescribed? This approach is validated [25] and was used to assess opioid craving [26-28].

#### Pain Intensity

Pain intensity was assessed using the 11-point verbal pain rating scale. The validity of the verbal pain rating scale is well established [29-31].

#### Michigan Body Map

The Michigan Body Map (MBM) is a self-report measure to assess body areas where pain is experienced. The MBM has demonstrated utility, reliability, and construct validity [32,33]. The MBM measure has been used to assess pain in a broad range of clinical settings [34-41].

#### Pain-Related Psychosocial Functioning

The Multidimensional Pain Inventory (MPI) measures the psychosocial impact of chronic pain [42]. The MPI has proven reliability and construct validity [43].

#### Negative Affect

Depressive symptoms and pain catastrophizing are key components of negative affect [44]. The Center for Epidemiologic Studies-Depression scale provides a validated measure of depressive symptoms [45] in patients with chronic pain [46,47]. The Pain Catastrophizing Scale provides a measure of negative cognitions and emotions associated with actual or anticipated pain experiences [48].

#### Baseline Smoking Status

Baseline smoking status was assessed using the techniques used by the Behavioral Risk Factor Surveillance System: (1) Have you ever smoked a cigarette, even a puff? (Yes or No); (2) Have you smoked at least 100 cigarettes in your entire life? (Yes, No, or Not sure); and (3) Do you now smoke cigarettes every day, some days, or not at all? (Every day, Some days, or Not at all) [49].

#### Quantitative Sensory Testing

Heat pain perception was quantified using the Computer Aided Sensory Evaluator IV (WR Electronics). This quantitative sensory testing device is validated [50-52], and we have used it to quantify opioid-induced hyperalgesia [15,16] and to study other pain-related states associated with altered heat pain perception [53-56].

#### Concomitant Treatments

Use of concomitant treatments for pain were assessed (eg, nonopioid medications, supplements, acupuncture, physical therapy, and chiropractic).

#### Sample Size Estimates

Based on our preliminary work [3,9,57-62], we estimated the rate of episodic opioid use would be 20% and the estimated rate of long-term use would be 7%. This would yield approximately 200 (780 × .27 = 210.6) patients developing some form of UPOU at each site. The analysis for specific aim 1 involved examining a wide variety of predictors of UPOU rather than 1 specific exposure. As a result, the minimum detectable odds ratios across a range of sample sizes were calculated. Setting the power at 0.8 and α at .05 (2-tailed), a total sample size of 1200 (600 cases and 600 controls) allowed detection of an odds ratio of 1.38 with a predictor that had a prevalence of 0.5 in controls and zero correlation in exposure between cases and controls. The detectable odds ratio (OR) goes up as the correlation between cases and controls becomes positive (detectable OR=1.48 with *r*=0.3), and it also increases as the prevalence of the exposure in the control group increases (detectable OR=1.55 with *r*=0.3 and prevalence of exposure=0.7 in controls).

#### Data Analysis Plan

The primary research question for specific aim 1 was to identify patient characteristics associated with UPOU. A case-control design was used with controls time matched to cases. The expectation was that multiple patient characteristics would be associated with UPOU. Descriptive statistics (means, medians, and ranges) were generated to compare cases and controls on all patient characteristics. Differences in patient characteristics between cases and controls were assessed using bivariate tests including the McNemar test for categorical variables and paired 2-tailed *t* tests (or Wilcoxon signed rank test, if needed) for continuous variables. The results of the bivariate analyses were used to select variables for conditional logistic regression analyses to regress UPOU on all variables identified as potentially associated with the UPOU. The regression models were constructed in stages by incorporating predictors in conceptual blocks and using standard selection methods to retain variables. All models included a fixed effect for site. ORs and 95% CI were used to evaluate the magnitude and statistical significance of the association between predictors and outcome.

### Specific Aim 2

#### Overview

Clinicians prescribing opioids to patients in specific aim 1 were recruited for assessment of prescribing characteristics.

#### Clinician Recruitment

Clinician recruitment was site specific, but a similar stepped approach was used. First, an email message was sent to each clinician briefly explaining that they had issued an opioid prescription to a patient participating in the observational study. The email note contained a link to a validated self-report survey regarding clinician attitudes and beliefs about opioids, and clinicians were asked to complete the survey [6]. Clinicians who did not respond were sent a reminder email 1 week later. Clinicians who did not respond to the reminder email after an additional 1-week period were sent a final email. After a 2-week period, all clinicians not responding to the email messages were sent a paper version of the survey using each site’s intracampus mail system. Clinicians not responding to the mailed survey after a 4-week period were considered nonresponders.

#### Study Assessment

Clinicians were assessed using the Clinicians’ Attitudes and Beliefs About Opioids Survey (CAOS) [6]. The validity and test-retest reliability of the CAOS has been reported [6]. The CAOS also assesses clinician demographics and practice characteristics including sex, age, years in practice, professional training (physician surgeon, nonsurgeon physician, nurse practitioner, and physician’s assistant), organization of practice (single specialty, partnership, solo, or multispecialty), practice structure (academic or university versus clinic or hospital based), proportion of weekly patient appointments involving management of chronic pain, and proportion of patients with chronic noncancer pain currently receiving opioids.

#### Sample Size Estimates

There was no information available to predict how many unique clinicians prescribe opioids to patients in aim 1. A 2013 meta-analysis of provider surveys estimated a survey response rate among health professionals of 53% [63]. Thus, we conservatively estimated the response rate would be between 35% to 65% and anticipated between 410 and 760 clinicians would complete the survey.

#### Data Analysis Plan

The primary research question for specific aim 2 was to identify clinician and practice environmental characteristics associated with UPOU. The analysis plan was similar to the plan described for specific aim 1. Descriptive statistics (means, medians, and ranges) were generated to compare cases and controls on all clinician and practice environmental characteristics. Differences in clinician characteristics between cases and controls were assessed using bivariate tests including the McNemar test for categorical variables and paired 2-tailed *t* tests (or Wilcoxon signed rank test, if needed) for continuous variables. The results of the bivariate analyses were used to select variables for conditional logistic regression analyses to regress UPOU on all variables identified as potentially associated with UPOU. The regression models were constructed in stages by incorporating predictors in conceptual blocks and using standard selection methods to retain variables. All models included a fixed effect for site. ORs and 95% CI were used to evaluate the significance of the associations between predictors and outcome.

### Specific Aim 3

#### Overview

Using the data generated in aims 1 and 2, SEM was used to evaluate the associations between framework elements in each domain. Identified associations were further evaluated in a large national administrative claims database.

#### SEM Analysis

The primary goal of aim 3 was to combine the results from aims 1 and 2 and extend these by building a statistical model that synergistically integrated the three domains of the UPOU conceptual framework. The UPOU framework has a number of complexities that limit the use of regression including multiple correlated predictors and outcomes, unobserved constructs that are difficult to directly measure, and nesting of patients with clinicians who are nested within sites. SEM can estimate latent variables and include them as both exogenous and endogenous variables in the same model, use multiple observable and quantifiable indicators to estimate latent variables from the shared variability of these indicators, and accommodate multiple endogenous variables (ie, dependent variables or outcomes) in 1 model. Multilevel SEM can estimate latent variables at 1 or more levels (eg, patient and clinician levels) and appropriately test associations at and across these different levels for categorical and continuous outcomes.

The SEMs were developed in several steps. The number of measurement models were estimated for all constructs that potentially used latent variables. Descriptive statistics were examined including means, medians, SDs, ranges, measures of central tendencies, and normality of the distributions. Extensive preliminary analyses were conducted to evaluate possible inconsistencies in the data, outliers, and variations in the data, and clinically and statistically relevant cutoff points that may facilitate the analyses were identified. The primary outcome measure was UPOU, measured as a dichotomous variable of cases and time-matched controls. The predictors of interest were grouped into 3 domains: patient characteristics, practice environment, and clinician characteristics. Each of these domains contained a number of directly observable and directly unobservable constructs. Examples of directly observable variables in the patient characteristic domain included age, sex, and employment status. Examples of directly unobservable constructs in the patient characteristic domain included symptoms of opioid withdrawal, opioid craving, and the psychosocial impact of chronic pain. Analyses (eg, coefficient α, principal components analysis, and factor analysis) were conducted to determine how to optimally combine scale items into reliable measures.

After all measurement models were established, a comprehensive structural model describing the conceptual framework was developed. In the first step, models were fitted separately by domain. For example, a parsimonious model relating patient characteristics to UPOU was developed. This first step involved a model-testing approach to trim a saturated model containing all patient-level constructs to develop a final parsimonious model that maximally explained UPOU with the least number of parameters. The results from aim 1 analysis directly informed this stage, but models varied due to the use of latent variables and a different statistical model. The Akaike information criterion, Bayesian information criterion, and the root mean square error of approximation were used to guide decision model trimming and refinement. This process was conducted separately for all 3 domains. The second step combined the parsimonious, domain-specific models into an integrated model. This involved multilevel SEM with a patient-level outcome (eg, UPOU) and predictors at the patient level, the clinician level, and the practice environment level. This model started with the 3 parsimonious, domain-specific models but then was expanded to test for possible cross-level interactions. For example, the effects of pain management training on UPOU varied by patient age. The conceptual model, previous research, and empirical findings were used to guide the model-building process, particularly with regard to the direction of association and what interactions were tested. This was not a process for testing all possible interactions; rather, only specific indicated effect modifications were tested.

Missing data were evaluated to determine if the pattern was missing completely at random (MCAR) or missing at random (MAR). If missing data were MAR or MCAR, SEM techniques were used to estimate missing values using either full information maximum likelihood or multiple imputation. If missing data did not fit an MAR or MCAR pattern, other options were explored including pattern mixture models. To the extent possible, potential bias due to attrition and missing data over time were assessed. All analyses were conducted using Stata (version 15, StataCorp) or MPlus (version 7, Muthén & Muthén).

#### Sample Size Estimates

Common algorithms do not exist for assessing power for complex SEM; however, 3 aspects of sample size were considered. First, because of the iterative and complex nature of SEMs, it was important to verify that the sample size was sufficiently large to produce stable model estimates. There are simulation studies suggesting that for models of the size and complexity proposed, minimum sample sizes for model stability would likely be in the hundreds [64]. Second, comparing models requires a certain sample size. Published estimates of model degrees of freedom suggest that sample sizes in the hundreds would be sufficient to discriminate competing models [64]. Finally, given a set sample size, an expected detectable effect size was estimated. Setting statistical significance at =.05 (2-sided tests) and power at 80%, a sample size of 500, for example, would allow detection effects of approximately 0.13 SD units across time (ie, considered a small effect). Even accounting for losses due to missing data, which can become an issue when combining multiple sources of data at multiple levels, the proposed sample size of 1200 patients, and the smaller number of clinician and practice environments in which they were nested, should be sufficient to estimate stable, comprehensive models and detect relatively small associations between constructs.

#### National Administrative Claims Database Analysis

The data collected prospectively on clinicians and patients at the 3 clinical sites provided detailed data on patients and clinicians from varied settings but were necessarily limited in sample size and derived from institutions affiliated with academic medical centers. As a result, a large national claims database was used to explore the findings of this study in a much larger and more diverse population.

The OLDW is an open, collaborative research and innovation center founded in 2013 [65]. The core linked data assets include deidentified claims data for privately insured and Medicare Advantage enrollees and deidentified EHR data from a nationwide network of provider groups. The database contains longitudinal health information on enrollees and patients, representing a diverse mixture of ages, ethnicities, and geographical regions across the United States. The EHR data are sourced from provider groups and reflects all payers, including uninsured patients.

The OLDW was used to estimate a structural model similar to that used in the analyses of clinical data from aims 1 and 2. The latent factors representing clinician, patient, and environmental factors associated with patients developing OPOU were estimated using OLDW data, and the results were compared with those estimated in the clinical data. The OLDW contains beneficiary characteristics including gender, age, race and ethnicity, household income, and geography (ie, state, county, or zip code). For beneficiaries submitting health insurance claims, a broad range of information is available including pharmacy claims (ie, prescribing clinician, drug type and date dispensed, days of supply), clinician and facility claims (ie, Current Procedural Terminology 4 codes, *International Classification of Diseases* [*ICD*]-*9* or *ICD-10* procedure codes, and *ICD-9* or *ICD-10* diagnoses), dates and place of service, cost data (ie, charges and patient and insurance amounts paid), and clinician type and specialty. In addition, OLDW includes laboratory data for 30% to 40% of patients whose clinicians have contracted with laboratories that provide data to OLDW.

Many of the concepts to be tested with clinical data were approximated using administrative claims data. Diagnostic codes were used to measure comorbidities including depression, anxiety and substance use disorders. Procedure and diagnostic codes were used to infer pain etiology from surgery, trauma, and other causes. Place of service and clinician specialty codes were used along with prescription fill information to determine the source of opioid prescriptions. Patient geographic information and prescription fill information were used to determine regulations governing the use of prescription drug monitoring programs.

### Ethical Considerations

The Mayo Foundation Institutional Review Board served as the reliant international review board (IRB) of record for all participating institutions including Mayo Clinic, the University of Michigan, Yale University, and the University of Minnesota. Written informed consent, using a mobile platform, was obtained from all study participants before study participation. Use of the mobile platform to obtain informed consent and collect study outcomes was IRB approved (#18-010484). All patient-related research information was deidentified before data analysis. All patients were remunerated US $50 for the initial screening, US $150 for the first clinical assessment, and US $100 for the second clinical assessment.

### Reporting Guidelines

The reporting of study methods and results adhered to the Strengthening the Reporting of Observational Studies in Epidemiology (STROBE) guidelines [66].

## Results

### Study Recruitment

#### Overview

The research proposal was funded on January 3, 2019, and patient recruitment began on August 1, 2019. However, due to the COVID-19 pandemic, patient recruitment was slowed in March 2020 and halted in April 2020 for a 4-month period. Over the next 2-year period, patient recruitment was slowed or interrupted at each clinical site based on the regional impact and the individual health care system response to the COVID-19 pandemic. Compared with the more urban setting of the University of Michigan and Yale University, the impact of COVID-19 pandemic on the rural catchment area of Mayo Clinic was less disruptive and associated with fewer interruptions. As a result, the majority of patient and clinician recruitment occurred at the Mayo Clinic site. Due to the unanticipated effects of the COVID-19 pandemic on clinical research activities, two 6-month, no-cost extensions were granted by the funding agency, and all recruitment and data collection were completed by August 2024.

#### Patient Recruitment

Table 1 depicts the total number of cases and controls recruited at each site. The Mayo Clinic site recruited 83% (855/1030) of the cohort, the Yale University site recruited 14.3% (147/1030), and the University of Michigan site recruited 2.7% (28/1030). Data analysis of patient characteristics associated with UPOU has been completed and the manuscript is under active development.

**Table 1:** Patient recruitment by study site.

Study site	Patients, n (%)		
	Total (n=1030)	Cases (n=513)	Controls (n=517)
Mayo Clinic	855 (83)	422 (82.3)	433 (83.8)
University of Michigan	28 (2.7)	15 (2.9)	13 (2.5)
Yale University	147 (14.3)	76 (14.8)	71 (13.7)

#### Clinician Recruitment

A total of 148 clinicians completed the CAOS survey. The Mayo Clinic site recruited 146 (98.6%) clinicians, and 2 (1.4%) clinicians were recruited at the University of Michigan site. Recruitment of unique clinicians at the Mayo Clinic site was limited, in part, by clinicians who prescribed opioids to more than 1 patient. Due to unanticipated administrative barriers at Yale University, no clinicians were recruited at this site. The data analysis of clinician characteristics associated with UPOU has been completed and the manuscript is under active development.

#### SEMs and National Administrative Claims Database Analysis

The SEMs are under active development, and the results of the SEMs based on the case-control data will be submitted for publication in a peer-review journal. The administrative claims database from OLDW has been successfully retrieved and is actively being prepared for data analysis using the SEMs developed from the case-control data. The results of the SEM and OLDW analysis will be submitted for publication in a peer-review journal.

## Discussion

### Principal Findings

The anticipated results of this prospective, case-control study should provide ample data to understand how patient, clinician, and practice environment characteristics facilitate or impede development of UPOU. More specifically, multivariate logistic regression models will elucidate the individual influence of each domain (eg, patient, clinician, and practice environment) on UPOU and multilevel SEMs will provide an estimate of latent variables and test associations at and across these different levels for categorical and continuous outcomes. Widespread dissemination will be facilitated by the results derived from the broader analysis of the OLDW.

The results of this study will further build existing knowledge about the clinical characteristics associated with UPOU. For example, in a systematic review and meta-analysis of 33 studies comprised of 1,922,743 individuals, patient-level characteristics associated with UPOU after surgery included female sex; high school level education; previous mental health diagnosis of depression, anxiety, or posttraumatic stress disorder; cocaine or alcohol use disorder; tobacco use; and preoperative use of prescription opioids, antidepressants, benzodiazepines, or antipsychotic medications [67]. In addition, a history of back and neck pain, fibromyalgia, and migraine headache were associated with UPOU after surgery [67]. However, interpretation of these findings was limited by varying definitions opioid naivety and UPOU, clinical and statistical heterogeneity, and varying approaches for establishing baseline clinical characteristics. The imprecision of the meta-analysis was due, in part, to the use of large administrative and insurance claims data by studies included in the systematic review, which limited the number of studies that were available for inclusion in the subgroup analysis of patient characteristics. The results of our study will extend these findings by including surgical and nonsurgical patients and using uniform definitions of opioid naivety and UPOU. Finally, due to imprecision associated with establishing previous psychiatric diagnoses [68,69], the severity of current depressive symptoms, the presence of positive and negative affect, and the level of pain catastrophizing were included as study outcomes.

The clinical and research implications of the study results are important for 2 reasons. First, this study leveraged a conceptual framework and a case-control design to identify patient and clinician characteristics associated with UPOU. It is anticipated that the patient characteristics will have an immediate and significant impact on the clinical care of opioid-naive adults receiving appropriately indicated opioid prescriptions for short-term use. The identified clinician characteristics will provide the opportunity to develop and deliver targeted educational content to clinicians to raise awareness of UPOU. Second, development of SEMs and secondary testing in the much larger and more diverse cohort derived from the OLDW will facilitate the design of future research projects aimed at reducing development of UPOU.

### Limitations

The timeline of the research project was disrupted by the COVID-19 pandemic, but data collection was facilitated by no-cost extensions and leveraging the rural catchment area of Mayo Clinic. This produced an imbalance in the number of patients and clinicians recruited between the 3 clinical sites. While the overall target sample size was largely attained and therefore target statistical power was met, the imbalance potentially limited generalizability and precludes site-specific analyses for Yale University and University of Michigan. The opioid use status of cases and controls were based on the number of prescriptions issued by clinicians. As a result, discrepancies could exist between the number of prescriptions issued compared with the number of prescriptions dispensed by pharmacies. Finally, the CONSORT criteria were adapted to categorize opioid use 3 months following the initial prescription. Although it is possible that the status of opioid use could change over time, all patients were followed an additional 6 months to assess the stability of the opioid use category in cases and controls.

### Conclusions

Following completion of data analysis, the study results will be immediately available for widespread dissemination in clinical practice and for use in ongoing research projects. It is anticipated that EHR systems will be leveraged to identify at risk patients receiving an initial opioid prescription and to send alert messages to members of the care team. This preemptive approach could also be used to develop direct-to-patient educational materials to raise awareness of the risk factors for developing UPOU.

## Abbreviations

AOS: Analgesic Outcomes Study
ASSIST: Alcohol, Smoking, and Substance Involvement Screening Test
CAOS: Clinicians’ Attitudes and Beliefs About Opioids Survey
CONSORT: Consortium to Study Opioid Risks and Trends
CTSA: Clinical and Translational Science Awards
EHR: electronic health record
FDA: Food and Drug Administration
ICD: International Classification of Diseases
IRB: institutional review board
MAR: missing at random
MBM: Michigan Body Map
MCAR: missing completely at random
MPI: Multidimensional Pain Inventory
NIH: National Institutes of Health
OLDW: OptumLabs Data Warehouse
OR: odds ratio
REP: Rochester Epidemiology Project
SEM: structural equation modeling
STROBE: Strengthening the Reporting of Observational Studies in Epidemiology
UPOU: unintended prolonged opioid use

## Data Availability

The study data will be presented in the main results sections of peer-review journals and as supplemental files when indicated.
